# Development of a high-throughput method for the systematic identification of human proteins nuclear translocation potential

**DOI:** 10.1186/1471-2121-10-69

**Published:** 2009-09-22

**Authors:** Trinh Xuan Hoat, Nicolas Bertin, Noriko Ninomiya, Shiro Fukuda, Kengo Usui, Jun Kawai, Yoshihide Hayashizaki, Harukazu Suzuki

**Affiliations:** 1 RIKEN Omics Science Center, RIKEN Yokohama Institute, 1-7-22 Suehiro-cho, Tsurumi, Yokohama 230-0045, Japan

## Abstract

**Background:**

Important clues to the function of novel and uncharacterized proteins can be obtained by identifying their ability to translocate in the nucleus. In addition, a comprehensive definition of the nuclear proteome undoubtedly represents a key step toward a better understanding of the biology of this organelle. Although several high-throughput experimental methods have been developed to explore the sub-cellular localization of proteins, these methods tend to focus on the predominant localizations of gene products and may fail to provide a complete catalog of proteins that are able to transiently locate into the nucleus.

**Results:**

We have developed a method for examining the nuclear localization potential of human gene products at the proteome scale by adapting a mammalian two-hybrid system we have previously developed. Our system is composed of three constructs co-transfected into a mammalian cell line. First, it contains a PCR construct encoding a fusion protein composed of a tested protein, the PDZ-protein TIP-1, and the transactivation domain of TNNC2 (referred to as ACT construct). Second, our system contains a PCR construct encoding a fusion protein composed of the DNA binding domain of GAL4 and the PDZ binding domain of rhotekin (referred to as the BIND construct). Third, a GAL4-responsive luciferase reporter is used to detect the reconstitution of a transcriptionally active BIND-ACT complex through the interaction of TIP-1 and rhotekin, which indicates the ability of the tested protein to translocate into the nucleus. We validated our method in a small-scale feasibility study by comparing it to green fluorescent protein (GFP) fusion-based sub-cellular localization assays, sequence-based computational prediction of protein sub-cellular localization, and current sub-cellular localization data available from the literature for 22 gene products.

**Conclusion:**

Our reporter-based system can rapidly screen gene products for their ability to be translocated to the nucleus. Large-scale applications of the system presented herein should provide invaluable information for a more complete biological atlas.

## Background

Mammalian nuclei are extremely dynamic organelles. They are structured into domains and contain numerous distinct architectural features related to their function [1-3]. Macromolecules important for the cell nuclei are shuttled between the nuclear and cytosolic compartments under the direction of nuclear localization signals (NLSs) and nuclear exclusion signals (NESs) that are responsible for nuclear import and for nuclear export of proteins, respectively [4-8], through the nuclear pore complexes [9-12]. The NLSs and NESs are recognized by the nucleocytoplasmic transport factors. Most nucleocytoplasmic transport factors belong to the family of karyopherin β protein known as importin-β [13]. Importin-β is a complex protein carrier and acts as a transport factor for proteins carrying NLSs [14,15], as it is able to function either as a direct carrier or via an adapter protein binding to the typical NLSs of proteins [7]. NLSs are short regions with a high amount of the basic amino acids arginine, lysine, and proline [16,17]. The main classes of typical NLSs are (i) SV40-like NLSs PKKKRKV, which are composed of a single peptide region containing basic residues [16,18], (ii) the nucleoplasmin signal, which is composed of two peptide regions containing basic residues that are separated by ten residues [19], and (iii) the unusual KIPIK NLS, which can be found in the amino-terminal signal of the Mat α2 yeast protein [17,20].

Nuclear protein import in mammalian cells requires soluble cytoplasmic co-factors [7]. Importins associate with their macromolecular cargo in the cytoplasm. They directly or indirectly translocate to the opposite side of the nuclear envelope via NPCs and release their cargo. Most β-karyopherins bind their cargoes directly, and importin-β is able to recognize cargo substrates without the need for any adaptors [12]. However, in some cases, instead of binding directly to NLSs, importin-β binds to importin-α, which then binds the NLSs. The typical NLS is imported exclusively by importin-β in conjunction with members of the importin-α family. Many other import signal peptides are basic and are often part of protein domains that bind RNA and DNA, and these signal peptides can bind *in vitro *to different importins [8].

Important clues to the function of novel and uncharacterized proteins can be obtained by identifying the potential nuclear translocation of a protein [21]. In addition, a comprehensive definition of the nuclear proteome will undeniably represent a key step toward a better understanding of the biology of this organelle. This manuscript describes research conducted as a part of the FANTOM4 Project, in which the main goal was to decipher the transcriptional regulatory networks in nucleus underpinning monocyte differentiation [22]. The FANTOM4 project used a complete catalog of nuclear proteins derived from the literature. Although the FANTOM4 project uncovered key features of the transcriptional network, knowledge of a more complete and experimentally derived list of proteins being able to translocate in the nucleus will undoubtedly be of significant impact and reveal additional important interactions.

There are several high-throughput experimental screening methods used to examine the sub-cellular localization of proteins and their nuclear localization: gene trap screening [21], systematic *in situ *ORF (open reading frame) tagging mediated by oligonucleotide-directed homologous recombination [23], large-scale gene-tagging [24-26], and random cDNA-GFP fusions [27,28]. However, these experimental approaches focus mainly on the predominant localizations of proteins and transient translocation of a protein into the nucleus can easily be overlooked.

Here, we report the development of a reporter-based system to systematically analyze the nuclear translocation potential of proteins. Our system is based on a modification of our high-throughput mammalian two-hybrid system [29]. It has two key advantages: sample preparations are mediated by PCR and a quantitative luciferase reporter assay is used in lieu of a read-out. Those two features allow for the deployment of an analysis pipeline with enough throughputs to achieve a proteome-scale analysis of nuclear translocation potential.

## Results

### Development of the nuclear translocation assay

**Figure 1:**
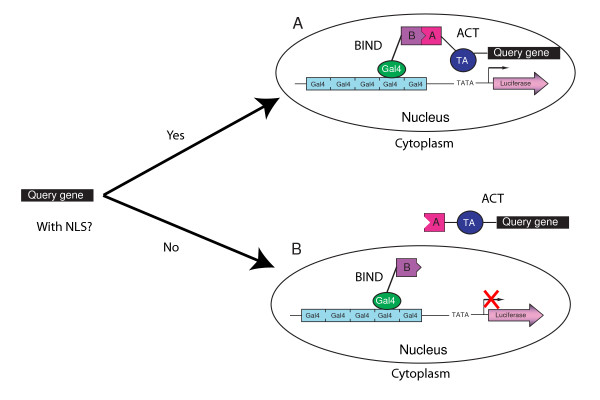
**Schematic representation of the system**. The pG5*luc *vector contains five GAL4-DNA binding domains upstream of a minimal TATA box, which in turn is upstream of the firefly luciferase gene. Box A and B are the interacting partners in ACT and BIND construct, respectively. Box TA in the ACT construct is a transcriptional activation domain. (A) If the target gene contains a NLS, the ACT construct product can translocate into the nucleus and interact with the BIND construct product, which activates the luciferase reporter gene. (B) If the target gene does not contain any coding sequence for NLSs, then the ACT construct product can not enter the nucleus and the luciferase gene remains inactivated.

We have developed a high-throughput assay to systematically identify a protein's potential for nuclear translocation according to the level of luciferase reporter activity (Figure 1). Our system is composed of three constructs. The first construct, ACT, encodes for a transactivation domain (TA) that is fused with the coding sequence of a domain A and a coding sequence (CDS) that we test for its ability to translocate to the nucleus. The second construct, BIND, encodes for a GAL4-DNA binding domain that is fused with the coding sequence of a domain B. The fusion proteins encoded in the ACT and BIND constructs can interact with each other via the selected interacting domains A and B. The third construct, a pG5*luc *vector containing five GAL4-DNA binding sites upstream of a minimal TATA box, which drives the expression of the luciferase (*luc*+) gene, acts as the reporter for the interaction between ACT and BIND constructs. The Gal4 DNA binding domain sequence used in the BIND construct contains a NLS that is sufficient for GAL4 nuclear localization [30-32]. Therefore, the fusion proteins generated by the BIND construct are constitutively able to enter the nucleus. We designed our system so that translocation of the fusion protein encoded by the ACT construct depends on the presence of a NLS in the target CDS; we have carefully engineered the interacting domain A and the transactivation domain TA which are capable of activating expression of the luciferase reporter gene and do not possess any localization signals. Therefore, the domain A::TA::CDS fusion protein is able to enter the nucleus only if the target CDS contains one or several NLSs. It interacts with BIND via the interacting-partners pair and reconstitutes an active GAL4 transcription factor that will induce the expression of the luciferase reporter gene (Figure 1A). On the other hand, the luciferase reporter gene will not be induced if the CDS lacks motifs encoding for NLSs (Figure 1B).

### Optimization of interacting partners in ACT and BIND constructs

A key feature of the system is the interaction of the ACT and BIND fusion proteins in the nucleus via domains A and B. This interacting pair, A and B, must satisfy the following criteria: 1) their interaction is well-characterized, 2) both domains are as small as possible so as not to be a limiting factor for the generation of fusion protein constructs containing large investigated CDSs, 3) the interaction is easily detected by the luciferase reporter expression, yet its affinity is weak enough that the ACT fusion proteins are seldom transported into the nucleus by associating with the BIND protein, 4) domain A does not possess any NLSs, and 5) domain B does not possess transactivation activity.

Satisfying criteria 1 and 2, we selected TIP-1 and rhotekin as the domains A and B, in which the reported interaction is mediated by the small domains, the PDZ domain of TIP-1 and the C-terminus sequence of rhotekin [33]. Further, the interaction affinity between the PDZ domain and its binding peptide has been reported as relatively weak (KD around 10^-7 ^M) [34]. We independently confirmed this interaction with the mammalian two-hybrid system from which the method reported herein is derived [35]. After we confirmed that the GFP-TIP-1 expression in mammalian cells is not localized in the nucleus (data not shown), we decided to further tailor rhotekin. Using the mammalian two-hybrid system, we tested a series of GAL4 DNA binding domain::rhotekin mutants fusion in which progressive deletion of rhotekin N-termini, Rhot443aa, Rhot257aa, Rhot111aa, and Rhot20aa were co-transfected with VP16 transactivation-TIP-1 fusion and the luciferase reporter plasmid into CHO-K1 cells. GAL4-Rhot20aa (remaining of the 20 last amino acids) was the optimal choice because we could maximize the signal resulting from the interaction with TIP-1 and minimize the background signal noise (detection of luciferase in the absence of an interacting partner; data not shown).

### Selection of the transactivation protein

We selected a transactivation domain (TA) to fuse to the TIP-1 PDZ domain that would 1) result in a small fusion protein and not interfere with the translocation potential of the added CDS, 2) possess a strong transactivation activity inducing the expression of the luciferase reporter, and 3) not induce translocation to the nucleus except when fused with a tested CDS possessing a NLS. We turned to our previous protein-protein interaction work in which we had systematically screened for protein self-activity: that is, a protein that when fused to Gal4 DNA-binding domain is able to interact with the transcriptional machinery and induce the expression of the reporter gene in the mammalian two-hybrid system [29]. TNNC2 (troponin C type 2) appeared as the optimal choice as it fulfilled all of our requirements (data not shown).

### BIND construct and high-throughput ACT construct preparation

**Figure 2:**
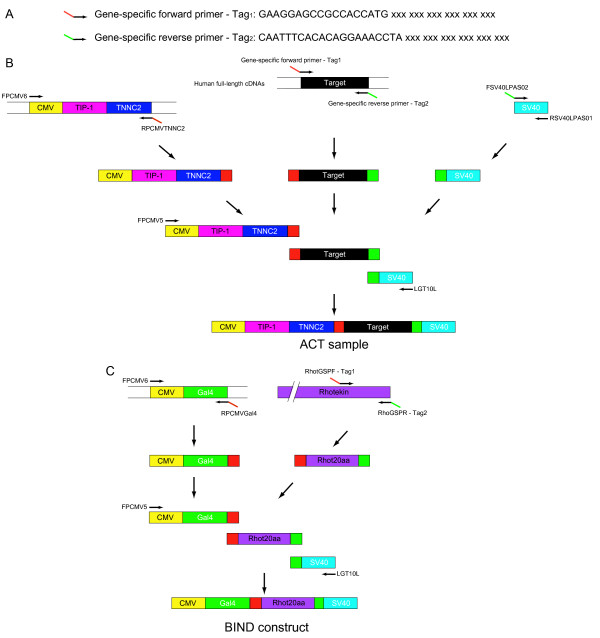
**Strategy for the high-throughput *in vivo *assay**. (A) Design of the gene-specific forward and reverse primers. The two common sequences Tag_1 _and Tag_2 _are used as margins to connect the cDNA with other DNA fragments. (B) Sample preparation. The gene-specific forward and reverse primers in (A) were used to amplify each targeted CDS. Red and green boxes are the two common sequences produced by Tag1 and Tag2 during PCR. The DNA fragments for CMV-TIP-1-TNNC2 and SV40 were obtained from the pACT vector. The PCR products were connected with the DNA fragments for CMV-TIP-1-TNNC2 and SV40 using FPCMV5 and LGT10L primers (ACT sample). (C) BIND-construct preparation. The DNA fragment for CMV-GAL4 was amplified from the pBIND vector using FPCMV6 and RPCMVGAL4 primers. A region of 20amino acids at the C-terminus of Rhotekin molecule was mediated and connected to the DNA fragments for CMV-GAL4 and SV40 (BIND construct).

Each ACT construct bearing a CDS of interest was created by a two-step PCR reaction. The CDS of each target gene was amplified with specific forward and reverse primers (Figure 2A) that produce two common sequences Tag_1 _and Tag_2 _at 5'- and 3'- terminus, respectively (red and green boxes in the first PCR products in Figure 2B). We also generated two common resources of PCR-amplified flanking fragments: the first one containing CMV-TIP-1-TNNC2 and the second one containing a SV40 poly-adenylation site (Figure 2B). Both resources of common DNA fragments were purified prior to use. Next, those PCR products were directly subjected to an overlapping PCR where the two common tag-derived sequences were used as margins to connect the DNA fragments of CMV-TIP-1-TNNC2, the target gene, and SV40 (Figure 2B). This two-step PCR reaction is performed without any intermediate purification steps, which further enhances the throughput of large collection preparations. The length of the PCR products was confirmed by 1% agarose electrophoresis (see Additional file 1). Using this approach, we could successfully amplify ACT constructs of up to 4.0 kb.

To generate BIND constructs, we employed a similar strategy; the DNA fragments for CMV-Gal4, and SV40 were amplified from the pBIND vector, purified, and used in an overlapping PCR to connect the DNA fragments of CMV-Gal4, Rhot20aa, and SV40 (Figure 2C).

### Selection of cells and conditions for the assay

**Figure 3:**
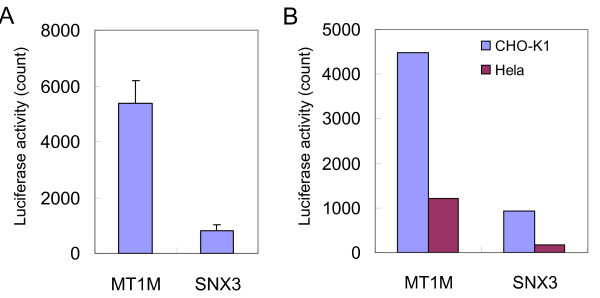
**Selection of cells using a nuclear localized protein, MT1M, and a non-nuclear protein, SNX3**. Reported values are luciferase activity; the error bars represent standard deviations. (A) MT1M and SNX3 were used to test the system using CHO-K1 cells. (B) Influence of cell lines was explored using hamster CHO-K1 cells and human HeLa cells.

To test if CDSs of interest can translocate to the nucleus, we rely on the detection of the interaction between TIP-1 and Rhotekin (fused to the queried CDS), both of which can be expressed only transiently. Thus, the assay only requires the transfection of PCR products, which is a process that is easily automated and systematic. As a proof of concept, we tested the system using MT1M, a metallothionein protein annotated to predominantly localize in the nucleus, and SNX3, a member of the sorting nexin family involved in cytoplasmic trafficking of proteins. The ACT, BIND, and luciferase reporter constructs were transfected into the CHO-K1 cell line using lipofection. As we expected, we found that MT1M containing ACT constructs induced high reporter activity, while the induction of the luciferase reporter gene was marginal for the ACT construct containing SNX3 CDS (Figure 3A).

Next we explored whether the type of cell line in which we performed our assay influenced the results. The ACT constructs for MT1M and SNX3, together with the BIND and luciferase reporter constructs, were transfected into the same number of CHO-K1 and HeLa cells. We observed that MT1M shows higher luciferase activity than SNX3 in both cell lines although CHO-K1 cells shows higher luciferase counts than HeLa cells (Figure 3B). Thus, the use of non-human mammalian cell line (CHO-K1) did not seem to impair the *in vivo *assay, and we decided to use CHO-K1 cells for further analysis.

Large proteins generally translocate to the nucleus more slowly than smaller ones. We therefore evaluated the adequacy of incubating for 20 hours post-transfection before lysis of cells in the luciferase reporter assay (see Additional file 2). We selected three coding sequences representative of a wide range of protein sizes: CRIP1 (77 aa), NANOG (305 aa), and ARNT2 (717 aa), and estimated their translocation after incubation for 20, 30, and 40 hours. We did not observe any significant differences in the read-out intensities or ratios for any of the three sampled coding sequences, suggesting that 20 hours of incubation is sufficient for obtaining a robust luciferase reporter gene activation even for large coding sequences.

**Figure 4:**
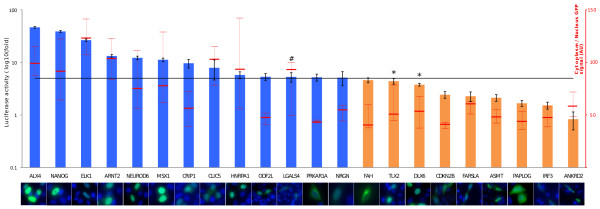
**Small-scale validation of the assay**. Luciferase-based nuclear translocation assay and GFP-fusion nuclear localization assay were compared for 22 constructs. Histogram represents the log10 of the average luciferase ratio for three independent assays. Error bars are standard deviation. The black line represents the 5-fold threshold above which a given construct is qualified as able to translocate into the nucleus; histograms in blue highlight positive luciferase results and those in orange negative results. The (#) and (x) signs, respectively, highlight the false-positive and false-negative results when compared to GFP-fusion-based nuclear localization. A representative picture of the GFP-fusion assay with blue DAPI straining and green GFP is positioned under each tested construct. The red line and error bars represent the ratio of GFP intensity in the nucleus to that of the cytoplasm computed from the GFP-fusion-based nuclear localization images. Values are also summarized in Additional File 4.

Next, we investigated if the presence of a strong nuclear exclusion signal affected the assay read-out (see Additional File 3). We made artificial constructs in which we fused the nuclear export sequence (NES) of the protein kinase inhibitor α (PKIA) to the carboxy terminus of two coding sequences that are able to be translocated to the nucleus (according to our luciferase reporter assay): NANOG and ELK1 (Figure 4 and Additional File 4). We then measured and compared the nuclear translocation of each of those two nuclear protein fusions to their respective PKIA NES fusion counterparts. The addition of the strong PKIA NES did not affect the nuclear translocation of NANOG. In contrast, the addition of PKIA NES to the carboxy terminus of ELK1 resulted in a drastic decrease in the luciferase ratio compared to that obtained with the native ELK1 ACT construct. The analysis of the sub-cellular localization of the GFP fusion version of those constructs corroborated the results of our luciferase-based reporter assay. Together, those results showed that our assay, as well as the GFP-fusion based assays, may be affected by the balance between the nuclear localization signal and the nuclear export signal of any given sequence.

### Small-scale validation of the assay

To test the ability to detect the translocation of proteins in the nucleus, we analyzed two sets of genes with nuclear localization reported in HPRD [36]. The first set was composed of 12 genes annotated as nuclear proteins (ALX4, IRF3, NANOG, MSX1, ELK1, NEUROD6, TLX2, DLX6, PAPOLG, ARNT2, ANKRD2, and HNRPA1) and the second set was composed of 10 genes annotated as cytoplasmic proteins (ASMT, FAH, FARSLA, ODF2L, PRKAR1A, NRGN, CRIP1, CDKN2B, CLIC5, and LGALS4). For each gene in those two sets, we performed the nuclear translocation assay in triplicate and conducted sub-cellular localization experiments by generating GFP-fused proteins. The gene-specific primers used to generate the 22 GFP constructs for the sub-cellular localization experiments were similar to those used to fuse the first PCR products of our luciferase reporter system. We then compared the results obtained from our luciferase reporter assay with our GFP sub-cellular localization experiments, HPRD annotation, and sequence-based sub-cellular localization *in-silico *predictions (PSORT II [37]) (Figure 4 and Additional file 4).

We considered assays reporting an average 5-fold ratio of the luciferase signal with the BIND construct co-transfected to luciferase signal without the co-transfected BIND construct to represent confident nuclear translocation potential, based on empirical results. Eight of the 22 genes were observed exclusively in the cytoplasm, 5 were exclusively in the nucleus, and 9 were diffusively localized both in the cytoplasm and the nucleus when GFP fusions were transiently expressed in CHO-K1 cells. Our mammalian two-hybrid derived assay was designed to detect the nuclear translocation potential of a CDS; therefore, we considered GFP sub-cellular localization assay reporting diffuse localization of encoded fusion protein both in the cytoplasm and the nucleus to represent true positive results. Though the luciferase ratio was 5.30 (± 1.08), LGALS4-GFP fusions localized exclusively in the cytoplasm. Therefore, compared to the GFP sub-cellular localization assay, the false-positive rate was 7% (1/13). Reciprocally, while DLX6 and TLX2 appeared to be located in the nucleus when fused to GFP, the luciferase ratios of those two genes were only 3.77 (± 0.42) and 4.4 (± 0.53), respectively. As a result, we can conclude that compared with a GFP sub-cellular localization assay, our system performed with a false-negative rate of 22% (2/9).

We also used the program PSORT II to predict the sub-cellular localization of those 22 genes and compared the most probable localization reported by the program to our assay. Again, although we predicted from the results of our assay that DLX6 and TLX2 are unable to translocate to the nucleus, PSORT II predicts them to be nuclear proteins, thus yielding a 22% (2/9) false-negative rate when compared with computational predictions. Four proteins with luciferase ratios ranging from 5.2 (± 0.79) to 7.9 (± 2.27) were predictd by PSORT II to be cytoplasmic proteins which results in a false-positive rate of 30% (4/13).

Sub-cellular localization annotations reported in HPRD agreed poorly with our assay. Under the 5-fold luciferase signal threshold that we used to define proteins as able or not to translocate to the nucleus, our comparison of the reporter-based system with the HPRD annotations showed a 46% (6/13) false-positive rate and a 55% (5/9) false-negative rate. It is important to note that this poor false-positive rate was in large part due to proteins for which our assay gave results that were very close to the 5-fold threshold we defined; 4 out of 6 false-positive results arose from luciferase assay in the 5.13 (± 1.54) to 5.33 (± 0.8) range. Thus, under a stricter definition of the cut off for which a protein is considered to be able to translocate into the nucleus, comparisons of our assay to HPRD annotations would result in a more reasonable 14% false-positive rate. Additionally, our observations of CRIP1 nuclear localization in the GFP-fusion and luciferase reporter-based assays as well as PSORT II prediction contrasts with the lack of nuclear annotation noted for CRIP1 in HPRD. Similarly, the relatively high false-negative rate can be counter-balanced by the observation that 1) both ANKRD2 and IRF3 that were also consistently predicted by our luciferase assay, our GFP fusion assays, and PSORT II as not localized in the nucleus and 2) TLX2 and DLX6 were also mistakenly characterized in our assay when compared to our own GFP-fusion assay.

Finally for each of the 22 GFP fusions, we conducted a quantitative analysis of the distribution of GFP signal located over the nuclear versus that distributed in the cytoplasm. For 5 to 7 single-cell images per construct, the DAPI and GFP signals were used to locate, respectively, the nucleus boundary and the extent of the cytoplasmic compartments. The average intensity of GFP within the nucleus boundary was then computed and compared to that of the cytoplasm. A good correlation between those GFP signal intensity ratios and luciferase activities was observed, providing yet another line of evidence that the luciferase activity measured in our assay accurately reflects the nuclear translocation potential of a particular coding sequence (Figure 4 and Additional file 5).

To test the capacity of our method to detect the translocation potential of proteins located in the cytoplasm during steady state but known to shuttle between the nucleus and the cytoplasm, we selected three known cases and assayed their nuclear translocation: GTSE-1 [38], dishevelled/DVL2 [39], and survivin/BIRC5 [40] (see Additional File 6). We could accurately predict the nuclear translocation potential of GTSE-1 and disheveled, yielding an average luciferase ratio of 9.98 and 9.88, respectively. On the other hand, the average luciferase ratio obtained for BIRC5 was only 2.24. A possible explanation for the failure to detect survivin/BIRC5 translocation potential could be the loss of its anti-apoptotic property upon nuclear localization [41].

## Discussion

The assay described here can be used to systematically characterize human gene products ability to translocate in the nucleus. It is easy to prepare samples by designing the gene-specific forward and reverse primers, in which no purification or cloning steps are also required The assay uses a luciferase reporter to directly and quantitatively measure if the easily engineered hybrid protein is able to undergo nuclear translocation. Since both the PCR-based sample preparation and the luciferase-based reporter assay can easily be manipulated in 96- or 384-well plate formats, we believe that our system can achieve the throughput required for a proteome-scale analysis of nuclear translocation potential. We demonstrated the validity of our approach using HeLa and CHO-K1 cells, but as long as lipofection of PCR products is tolerated, a wide range of alternative cell types can be employed.

While our luciferase reporter system gave results relatively consistent with those obtained by GFP fusion assay, our results did not correlate well with literature-derived localization reported in HPRD. Perhaps protein localizations that are too often reported in the literature are limited to the predominant localizations of a protein, with minor sub-cellular localizations often hardly accessible to search for location and therefore are poorly described. In addition, methods relying on ectopic protein over-expression to report sub-cellular localization may overlook minor localizations of proteins, or even induce unusual sub-cellular localization. On the other hand, a fusion with TIP-1 PDZ domain and TNNC2 trans-activation domain, which our system relies on, may also hamper the genuine localization of the protein. In some cases, the binding of rohtekin to the TIP1 PDZ domain could be altered by the fusion of particular CDS, thus preventing the reconstitution of a transcriptionally active BIND-ACT construct and the detection of the effective translocation of ACT in the nucleus by the luciferase reporter gene. This scenario is probably the most plausible reason for failing to detect the nuclear translocation of TLX2.

Methods used to characterize sub-cellular localizations of proteins usually focus on a description of cellular compartments where the proteins are predominantly localized. On the other hand, our system can provide information concerning the localization or function of a gene product that is not apparent from previous studies [42]. Since this system is able to report the potential nuclear translocation potential of any given protein-coding sequence, it allows for a much more thorough cataloging of the mammalian nuclei proteome. Such a comprehensive parts-list is a key element for deciphering the biology of such an extremely dynamic organelle such as the nucleus. The nuclear translocation data accumulated can be cross-referenced with the static protein-protein interaction network or gene expression atlas. Our assay also has the advantage of having a quantitative read-out. In order to compare the continuous values reported to annotations derived from our GFP-fusion assays, computational predictions, and literature-derived annotations, we have performed discretization of its outcome and empirically chosen a 5-fold ratio of the luciferase signal with the BIND construct co-transfected to the luciferase signal and without the co-transfected BIND construct to confidently represent potential nuclear translocation. It is important to note that this minimum fold ratio threshold remains open to refinement, in particular in the light of further experiments with proteins for which the sub-cellular localizations are richly documented. Finally, our assay also offers unprecedented potential for scaling up and analysis of nuclear translocation potential under different cellular conditions such as drug treatment, knockdown-mediated silencing, or conversely over-expression of genes involved in nuclear translocation. We also expect our system to be quite valuable for the identification of novel localization sequences in proteins that translocate to the nucleus but have atypical NLSs.

## Conclusion

We have described a new method of analyzing the nuclear translocation potential of a given coding sequence. Our method can easily be employed in parallelized settings to analyze nuclear translocation potentials upon different cellular conditions and treatments. The major advantages of the method are its ease of use and the scalability of both sample preparation and final read-out. Since the PCR-based sample preparation and the luciferase-based reporter assay can be used in 96- or 384-well plates, we believe that our system can achieve the throughput required for proteome-scale analysis.

## Methods

### PCR primers

Gene-specific forward and reverse primers for amplification of target genes were designed as described previously [29]. Other primers are indicated in Additional File 7.

### Constructs and assay samples

PCR procedures were performed as described previously [29,43]. To generate the BIND construct, we amplified DNA fragments for CMV, GAL4-DNA binding domain and for SV40 poly-adenylation signal with BIND vector (Promega), then purified them with Wizard^® ^SV Gel and PCR Clean-up System (Promega) before the second PCR. In addition, a fragment of 20 amino acids at the C-terminus of Rhotekin was also generated using a set of primers Rhot20aaF and RhotR. The second PCR was carried out to connect the DNA fragments for CMV-GAL4, Rhot20aa and the SV40 poly-adenylation signal. TIP1 CDS and TNNC2 CDS were cloned into multi-cloning sites of pACT vector (Promega) to mediate the ACT construct. The DNA fragment for CMV-TIP1-TNNC2 was subsequently amplified with FPCMV6 and RPCMVTNNC2 and purified as described above. The CDS of each human cDNA was amplified with the corresponding gene-specific forward and reverse primers (the first PCR) and directly subjected to the second PCR. The fragments for CMV-TIP1-TNNC2, CDS and SV40 poly-adenylation signal were connected by the overlapping PCR using a primers-set FPCMV6 and LGT10L. All of the PCR products were confirmed by agarose gel electrophoresis.

### Sub-cellular localization assay using luciferase reporter

Sub-cellular localization assay was carried out in 384-well assay plates and the M2H assay was assayed as described previously [29,43] with the following modification: 1/400 dilution of the BIND construct diluted in culture medium was co-transfected with individual ACT constructs into mammalian cells. Each ACT sample was diluted 20 times, then 4 μl was mixed with 4 μl of the diluted BIND construct in 10 μl of culture medium, Opti-MEM (Invitrogen). Next, 21.24 ng of pG5*luc *vector was added to the mixture and 8 μl of the transfection reagent Lipofectamine™ 2000 (Invitrogen), which was diluted 50 times in culture medium, was added to the mixture and mixed gently before incubation at room temperature for 20 minutes. Fourteen microliters of cells (1.6 × 10^6 ^cells/ml) was mixed well with the mixture and samples placed into each of the 384 wells. The samples were incubated at 37°C for 20 h in a CO_2 _incubator. The luciferase activity was measured with the Steady-Glo luciferase assay system (Promega) and Wallac ViewLux 1430 UltraHTS MICROPLATE IMAGER (PerkinElmer Life Science). Each assay was performed in triplicate and the final result was the average, n = 3.

### Protein sub-cellular localization using GFP fusion proteins

To construct a model for sub-cellular localization experiments, we first amplify the fragment CMV-EGFP-Tag_1 _with CMV_GFP1 and pEGFP-C1-CMVR-Tag_1 _primers, and the fragment for Tag_2_-SV40 with Tag_2_-pEGFP-C1-SV40F and SV40_GFPRev1 primers from the plasmid pEGFP-C1 (CLONTECH), where Tag_1 _and Tag_2 _are the two common sequences used to connect the DNA fragments during sample preparation (see the Figure 3 legend). We also PCR-amplified CDSs using specific forward and reverse primers, then subjected them to a second PCR amplification to connect CMV-EGFP-Tag_1 _with Tag_2_-SV40. To set up a positive control for cytoplasmic localization, we fused GAPDH with CMV-EGFP-Tag_1 _and Tag_2_-SV40. A fragment including CMV-EGFP-SV40 was PCR-amplified from the plasmid pEGFP-C1 as another positive control for cytoplasmic localization. To set up a positive control for nuclear localization, we amplified CMV-ECFP-(NLS)_3_-SV40 from the plasmid pECFP-Nuc (CLONTECH).

The PCR products were transfected into HeLa and CHO-K1 cells, then grown on 24-well plates for 20 h. Twenty-four hours after transfection, cells were washed with 1× PBS and fixed in 0.5 ml of 1× PBS (Sigma) containing 4% of paraformadehyde (Wako Pure Chemical Industries, Ltd) for 10 minutes at room temperature followed by a wash with 1× PBS three times. The cells were stained with 200 μl of twice pre-diluted of VECTASHILED Mounting Medium with diamidino-2-phenyl-indole (DAPI) (Vector Laboratories). Fluorescence images were acquired using the inverted research microscope DM IRE2 (Leica, Wetzlar, Germany) equipped with N PLAN L 20× 0.40 NA CORR (Leica) or PL Fluotar L 40× 0.40 NA CORR (Leica) lens. Image acquisitions were performed with Leica's FW4000 software. For each image, a 359 nm and a 490 nm wavelength fluorescence filter were used for DAPI and EGFP imaging, respectively. For these assays, a minimum of two independent transfections were performed.

### Quantitative analysis of GFP fusion protein sub-cellular localization

In order to quantify the nuclear localization of GFP fusion proteins, we wrote a custom Perl PDL script to analyze microscopic images of DAPI-stained, GFP fusion protein in transfected cells. For each fusion protein tested, 5 to 7 single cell images were delineated manually. For each image, we first applied a smoothening filter of 10 × 10 pixels on the DAPI signal, and selected the area corresponding to 1.5 deviations from the mean DAPI value as representative of the nucleus boundary. The rational behind this filtering process was confirmed by manual comparisons with direct cell imaging. A similar filter was applied to the GFP signal to delineate the extent of the area where GFP could be confidently detected. We then computed the average intensity of GFP within the nucleus boundary and compared it to that of the whole area delineated by the GFP signal. The reported values are the average ratios of the GFP intensity for each pixel of the single cell isolated images in arbitrary units.

## List of abbreviations

NLSs: nuclear localizations signals; NES: nuclear exclusion sequence; CDS: coding sequence; GFP: green fluorescent protein; PCR: polymerase chain reaction; TA: trans-activation domain.

## Authors' contributions

TXH, NN and SF produced the experimental data; NB and HS wrote the paper; JK, YH and HS managed the research. All the authors read and approved the final manuscript.

## Supplementary Material

Additional file 1 **Agarose gel electrophoresis of six ACT samples**. (A) CDSs of six representative target genes with various ORF length were amplified by gene-specific forward and reverse primers and confirmed on 1.5% agarose gel (the first PCR). λ-*Hin*dIII and ϕX-*Hae*III mix size marker was used. The expected bp sizes of 918 (*NANOG*), 2151 (*ARNT2*), 234 (*CRIP1*), 237 (*NRGN*), 417 (*CDKN2B*) and 1527 (*FARSLA*) were confirmed. (B) The first PCR products were directly applied to the second PCR to connect with the DNA fragments for CMV-TIP-1-TNNC2 and SV40 (ACT samples). The products were confirmed on 1.0% agarose gel (λ-*Sty*I marker). The expected bp size (ORF + 2.1 kb) was confirmed for each ACT sample. Click here for file

Additional file 2 **Effect of incubation time on the readout**. Three assay-positive constructs with various protein sizes (NANOG (305 aa), ARNT2 (717 aa) and CRIP1 (77 aa)) were selected from Additional File 4, then subjected to the assay with incubation times of 20, 30, and 40 h. Click here for file

Additional file 3 **Effect of NES addition on nuclear localized proteins**. (A) We fused two assay-positive proteins (NANOG and ELK1 in Additional File 4) with the nuclear export sequence (NES) of protein kinase inhibitor alpha (PKIA) at the carboxy terminus and conducted the assay. The results were normalized with those obtained with native proteins. (B) GFP-fusion assay of NANOG and ELK1 with/without the NES. Nucleus was stained by DAPI. Click here for file

Additional file 4 **Sub-cellular localization of the tested proteins in the in vivo assay**. Table summarizing the tub-cellular localization of the tested proteins in the in vivo assay Click here for file

Additional file 5 **Correlation between the ratio of GFP intensity in the nucleus to that of the cytoplasm computed from the GFP-fusion-based nuclear localization images and the log10 of the average luciferase ratio for three independent assays for 22 tested constructs**. Standard deviations are represented by the horizontal and vertical bars for the luciferase and GFP fusion quantitative analysis respectively. In blue the gene product that we detected as being able to translocate in the nucleus, in orange the gene product that did not translocate into the nucleus. Click here for file

Additional file 6 **Measure of the translocation potential of known steady state cytoplasmic proteins**. Selected steady state cytoplasmic proteins that can shuttle between the nucleus and the cytoplasm: GTSE-1, DVL2 and survivin/BIRC5. Reported values are average luciferase ratios for three independent assays. Error bars are standard deviation. Click here for file

Additional file 7 **List of primers used**. Primers name and sequence used in this study Click here for file
