# Supplementary figures and images for: Development of a high-throughput method for the systematic identification of human proteins nuclear translocation potential
Source: BMC Cell Biol. 2009 Sep 22;10:69. doi: 10.1186/1471-2121-10-69 (PMC2754447; doi:10.1186/1471-2121-10-69)

A

NANOG  
ARNT2  
CRIP1  
NRGN  
CDKN2B  
FARSLA

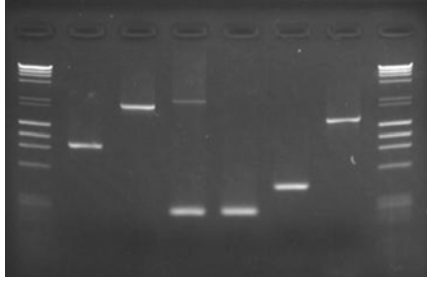

B

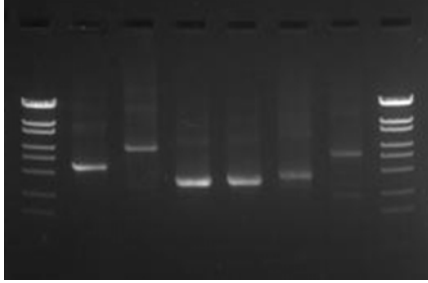

Supplement: Additional file 1 — Agarose gel electrophoresis of six ACT samples. (A) CDSs of six representative target genes with various ORF length were amplified by gene-specific forward and reverse primers and confirmed on 1.5% agarose gel (the first PCR). λ-HindIII and ϕX-HaeIII mix size marker was used. The expected bp sizes of 918 (NANOG), 2151 (ARNT2), 234 (CRIP1), 237 (NRGN), 417 (CDKN2B) and 1527 (FARSLA) were confirmed. (B) The first PCR products were directly applied to the second PCR to connect with the DNA fragments for CMV-TIP-1-TNNC2 and SV40 (ACT samples). The products were confirmed on 1.0% agarose gel (λ-StyI marker). The expected bp size (ORF + 2.1 kb) was confirmed for each ACT sample. [file 1471-2121-10-69-S1.PDF]

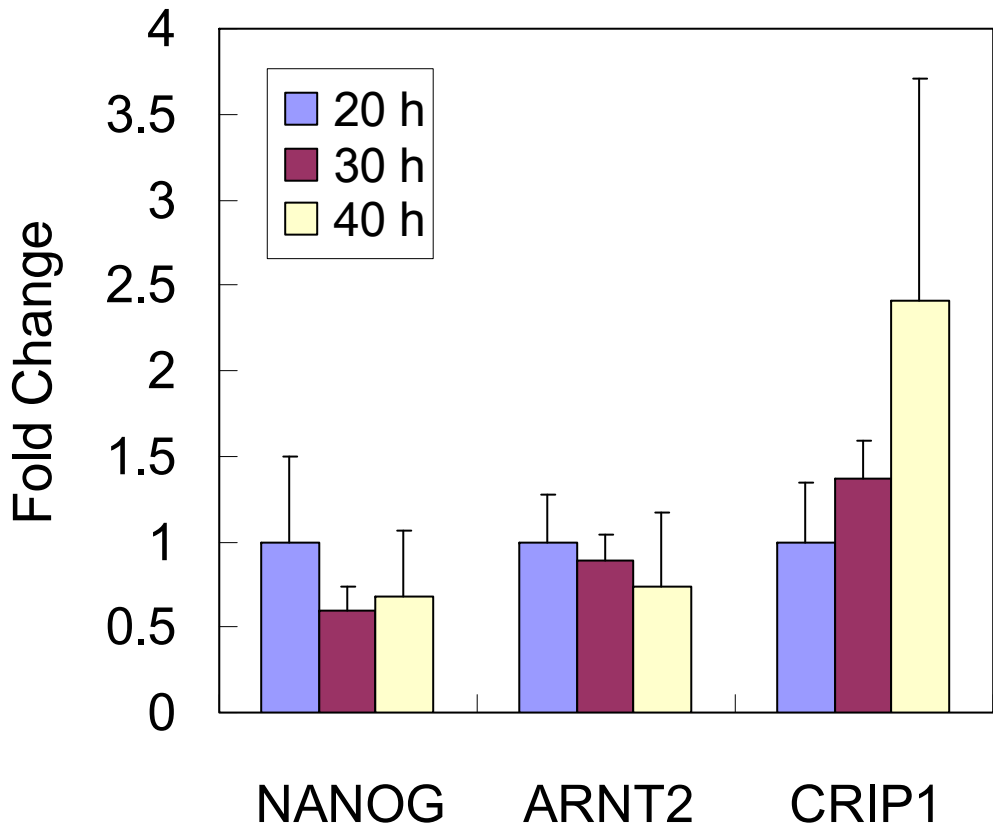

Supplement: Additional file 2 — Effect of incubation time on the readout. Three assay-positive constructs with various protein sizes (NANOG (305 aa), ARNT2 (717 aa) and CRIP1 (77 aa)) were selected from Additional File 4, then subjected to the assay with incubation times of 20, 30, and 40 h. [file 1471-2121-10-69-S2.PDF]

A

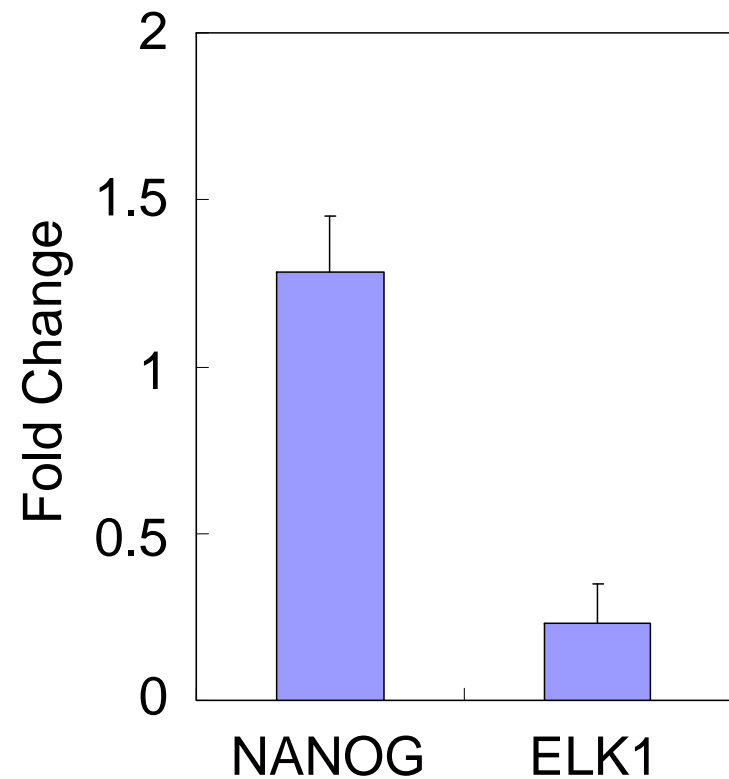

B

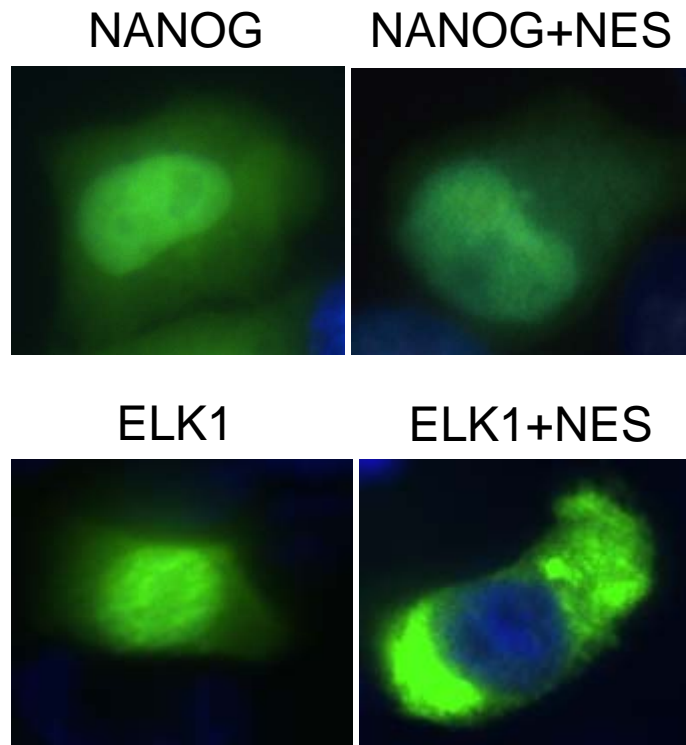

Supplement: Additional file 3 — Effect of NES addition on nuclear localized proteins. (A) We fused two assay-positive proteins (NANOG and ELK1 in Additional File 4) with the nuclear export sequence (NES) of protein kinase inhibitor alpha (PKIA) at the carboxy terminus and conducted the assay. The results were normalized with those obtained with native proteins. (B) GFP-fusion assay of NANOG and ELK1 with/without the NES. Nucleus was stained by DAPI. [file 1471-2121-10-69-S3.PDF]

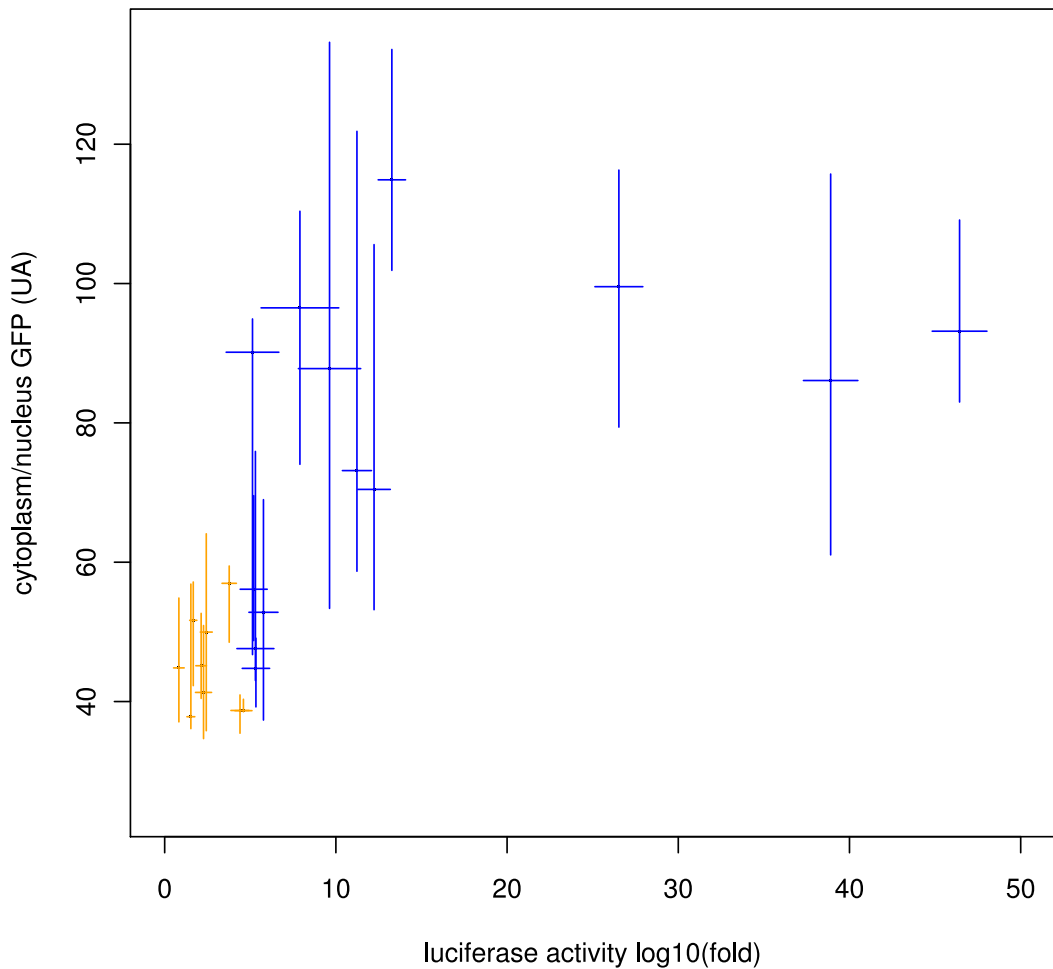

Supplement: Additional file 5 — Correlation between the ratio of GFP intensity in the nucleus to that of the cytoplasm computed from the GFP-fusion-based nuclear localization images and the log10 of the average luciferase ratio for three independent assays for 22 tested constructs. Standard deviations are represented by the horizontal and vertical bars for the luciferase and GFP fusion quantitative analysis respectively. In blue the gene product that we detected as being able to translocate in the nucleus, in orange the gene product that did not translocate into the nucleus. [file 1471-2121-10-69-S5.PDF]

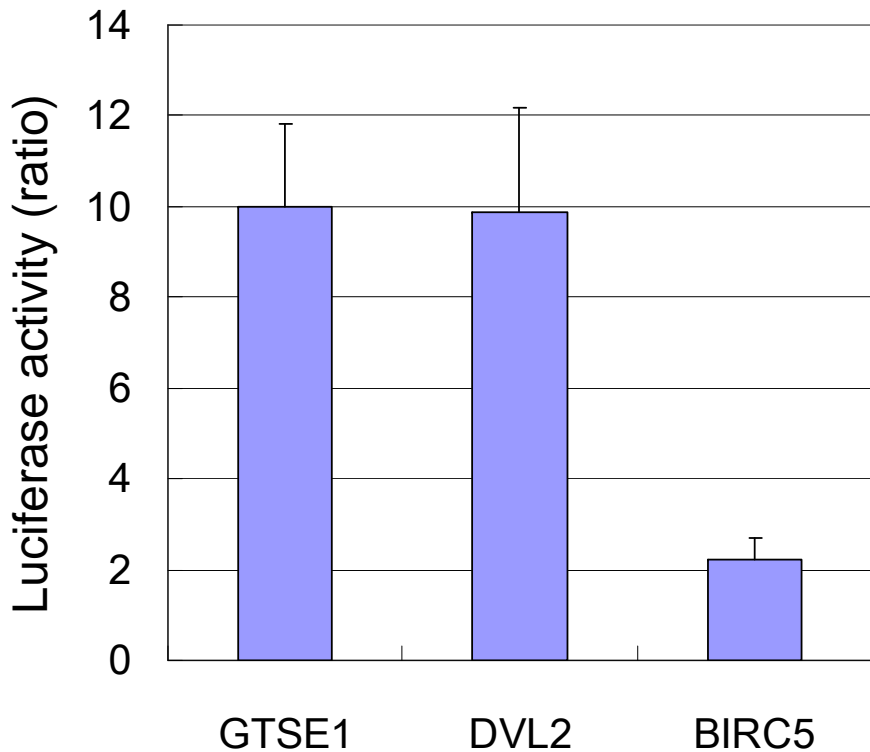

Supplement: Additional file 6 — Measure of the translocation potential of known steady state cytoplasmic proteins. Selected steady state cytoplasmic proteins that can shuttle between the nucleus and the cytoplasm: GTSE-1, DVL2 and survivin/BIRC5. Reported values are average luciferase ratios for three independent assays. Error bars are standard deviation. [file 1471-2121-10-69-S6.PDF]
